# Health-Care Utilization and Outcomes in Young Adults With Type 1 and Type 2 Diabetes

**DOI:** 10.1210/jendso/bvae115

**Published:** 2024-06-25

**Authors:** Anna Zenno, Alyssa Huang, Alissa J Roberts, Catherine Pihoker

**Affiliations:** Department of Pediatrics, University of Washington, Seattle, WA 98105, USA; Department of Medicine, University of Washington, Seattle, WA 98105, USA; Department of Pediatrics, University of Washington, Seattle, WA 98105, USA; Department of Pediatrics, University of Washington, Seattle, WA 98105, USA; Department of Pediatrics, University of Washington, Seattle, WA 98105, USA

**Keywords:** health-care utilization, clinical outcomes, young adults, type 1 diabetes, type 2 diabetes

## Abstract

Young adulthood can be a challenging time for individuals with diabetes mellitus (DM) as they experience increasing independence and life transitions, which can make it difficult to engage in DM self care. Compared to older adults, young adults are more likely to have higher glycated hemoglobin A_1c_ (HbA_1c_). They also often have lower adherence to standards of care in DM, and higher utilization of emergency department (ED) visits and hospitalizations for diabetic ketoacidosis. This review describes health-care utilization and explores factors that may contribute to high HbA_1c_ among young adults with DM. In addition, it discusses the unique health-care needs of young adults with DM, examines the role of technology in their DM care, and analyzes the effects of social determinants of health on their health-care utilization.

Diabetes mellitus (DM) is a chronic illness with increasing incidence and prevalence among youth in the United States and includes both type 1 diabetes (T1D) and type 2 diabetes (T2D) [1]. T1D often involves adhering to a complex and demanding medical regimen that requires frequent glucose monitoring, daily insulin administration, and dietary management with carbohydrate-counting for some on multiple daily injections of insulin. T2D can be similarly demanding, particularly for those treated with insulin or other intensive medical therapy and requiring frequent glucose monitoring. Individuals of all ages may struggle with adhering to their DM management, but young adults between ages 18 and 25 years are especially at high risk as they navigate a developmental period characterized by increasing independence and multiple life transitions, which can make it difficult to prioritize DM care [2]. This is reflected in data from the T1D Exchange electronic health-care record database, which revealed that among 12 035 adults with T1D, young adults in the 19- to 25-year-old age group had the lowest proportion of adults meeting the recommended glycated hemoglobin A_1c_ (HbA_1c_) target of less than 7% at 17.4%, compared to 31.5% among those aged 26 to 49 years, and 28.2% among those 50 years and older [3]. In addition, younger adults were more likely than older adults to have an HbA_1c_ greater than 9%, which is concerning given the strong association of elevated HbA_1c_ with higher rates of DM-related complications and emergencies [3].

This review explores factors that may contribute to the suboptimal glycemic control among young adults with DM, such as decreased adherence to DM standards of care and challenges of transitioning from pediatric to adult care. As a consequence, they face increased occurrence of acute DM complications, utilization of emergency department (ED) visits, hospitalizations for diabetic ketoacidosis (DKA), and early emergence of DM-related complications [4]. In addition, it discusses the unique health-care needs of young adults with DM, examines the role of technology in their DM care, and analyzes the effects of social determinants of health on their health-care utilization.

## Materials and Methods

**Figure 1.**
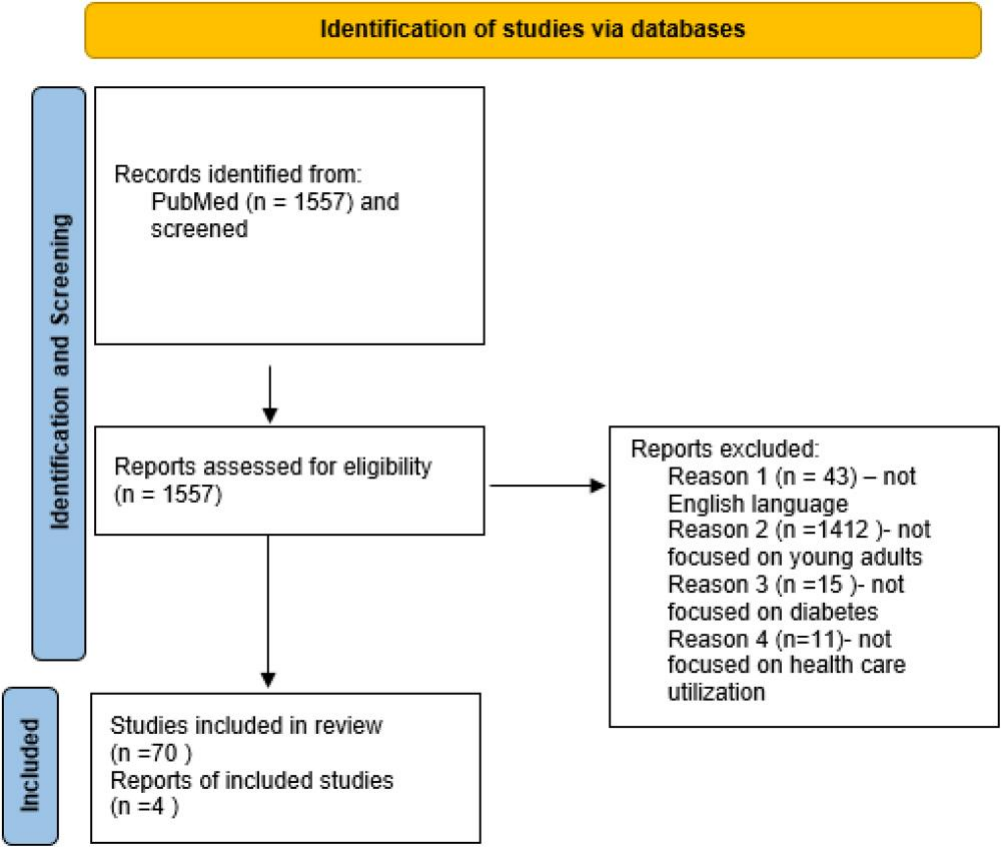
PRISMA flow diagram. *Used search terms “healthcare utilization” OR “hospitalization” OR “emergency care” OR “urgent care” OR “comorbidity” OR “medication” OR “pregnancy” OR “mental health” AND “type 1 diabetes” OR “type 2 diabetes” OR “diabetes” AND “young adults” OR “youth,” from 2003 to 2023.

We searched PubMed using the terms “*healthcare utilization*” OR “*hospitalization*” OR “*emergency care*” OR “*urgent care*” OR “*comorbidity*” OR “*medication*” OR “*mental health*” AND “*type 1 diabetes*” OR “*type 2 diabetes*” OR “*diabetes*” AND “*young adults*” OR “*youth*,” from 2003 to 2023. The initial search yielded n = 1557 items. Of these, 1485 were excluded because they did not discuss relevant health-care utilization, were not focused on young adults, were in a language other than English, or were not peer-reviewed (Fig. 1). Four additional references were identified by reviewing the reference lists of included articles, and subsequently added in the review if they were relevant.

## Standards of Care

The American Diabetes Association (ADA) updates and publishes standards of care annually. This section describes the adherence to these standards of care by young adults with DM.

### Quarterly Visit

The frequency of DM care for young adults with T1D and T2D can vary based on individual needs and glycemic control. In general, quarterly medical visits are recommended by the ADA [5]. The ADA also recommends that individuals with DM have an HbA_1c_ checked at least twice a year if they are meeting treatment goals and have stable glycemic control [5], whereas the International Society for Pediatric and Adolescent Diabetes (ISPAD) recommends all youth with DM have a minimum of 4 HbA_1c_ measurements per year (at ∼3-month intervals) [6].

Consistent use of a continuous glucose monitor (CGM) is an alternative tool health-care providers can leverage to assess the control of an individual's DM control by reviewing their CGM's glucose management indicator, which can be used as a proxy for serum HbA_1c_.

### Laboratory and Comorbidity Screening/Complications

To reduce risk of macrovascular complications, individuals with DM are recommended to undergo screening and management of cardiovascular risk factors such as hypertension and dyslipidemia. In addition, screening for microvascular complications associated with DM such as eye examinations for diabetic retinopathy, urine microalbumin testing for diabetic kidney disease, and foot examinations for diabetic neuropathy are recommended by ADA Standards of Care guidelines and ISPAD Clinical Practice Consensus guidelines [5, 6].

### Transition From Pediatric to Adult Care

Multiple studies have shown that young adults with T1D and T2D do not achieve target glycemic control and experience gaps in care prior to and during the period of health-care transition [7, 8]. Thus, youth with DM should begin preparation for transition to adult health care with guidance from their pediatric diabetes care team in early adolescence, and ideally at least 1 year before transition [5, 6]. Comprehensive and coordinated planning is crucial. Youth with DM may benefit from a multidisciplinary team that includes a medical provider, social worker, and psychologist to help them navigate what can be a challenging period for many youth. Diabetes transition interventions such as structured transition programs and appointment navigation have shown promising results [9, 10]. For example, the Helmsley T1D Transition Let's Empower and Prepare (LEAP) Program was a structured transition program with quarterly visits led by a multidisciplinary team consisting of a certified DM educator, registered dietitian, and medical provider in Los Angeles, California [9]. Unique benefits of the LEAP Program included the development of tailored DM education and the use of case managers who coordinated transfer from the pediatrics clinic to the adult clinic and encouraged adherence to scheduled visits. A prospective, nonrandomized trial that compared the efficacy of the LEAP Program to standard care showed that participants who participated in the structured transition program had improved glycemic control, incidence of severe hypoglycemia, global well-being, and increased success in transition to adult care at 12 months [9]. In addition, a randomized, open-label, controlled trial in Melbourne, Australia, investigated the effect of an appointment-management intervention consisting of personalized preappointment telephone and short message service (SMS; text) reminders with automatic rebooking of missed appointments on clinic attendance after transition and found that appointment management increased clinic attendance of young adults with T1D during 12 to 24 months after transition to adult care compared to the control group [10].

Multiple studies have reported that youth and young adults with DM tend to have better outcomes if their care provider is an endocrinologist, rather than a generalist [11]. Young adults who were not enrolled in a university were less likely to be independent in their DM care, and less likely to have a successful transition to an adult-oriented provider [12].

### Adherence to Diabetes Mellitus Standards of Care Guidelines

Adherence to ADA Standards of Care guidelines for retinopathy screening is poor, with only 26.3% meeting ADA recommendations among 2949 patients with T1D for at least 5 years without a history of diabetes retinopathy [13]. Another study showed that retinopathy screening occurred on time in only two-thirds of youth with T1D [14], with missing school or work being the most reported barrier. Other barriers include lack of knowledge (eg, symptoms, confusion between screening and standard eye check), poor geographic access, transportation difficulties, depression, and absence of a recommendation by the health-care provider [14, 15].

A cross-sectional survey of participants in the SEARCH for Diabetes in Youth study on self-report of meeting standards of DM care revealed that only 68% underwent HbA_1c_ testing and 66% underwent an eye examination in accordance with ADA recommendations [16]. Young adults aged 18 years or older tended to have fewer screening tests for DM complications regardless of DM type, but particularly with T2D. Only 45% of participants aged 18 years or older with T2D reported having their HbA_1c_ levels measured with the recommended frequency, compared with 70% of participants aged 12 to 17 years (*P* = .0009). Significant differences between the two age groups were also found with respect to lipid level testing (69% of participants aged ≥18 years with T2D vs 100% of participants aged 12-17 years), blood pressure measurements (82% of participants aged ≥18 years with T2D vs 96% of participants aged 12-17 years), and microalbuminuria testing (65% of participants aged ≥18 years with T2D vs 89% of participants aged 12-17 years). Among participants with T1D, significant differences between age groups were found for HbA_1c_ testing (55% for age ≥18 years vs 73% for ages 12-17 years and 80% for age ≤11 years; *P* < .0001) and for lipid-level testing (83% for age ≥18 years, compared with 96% for ages 12-17 years; *P* < .0001). In general, youth aged 12 to 17 years with DM had higher rates of self-reported screening than young adults aged 18 years and older. Older age (≥18 years) and lower family income (annual household incomes ≤$25 000) were associated with decreased adherence to guidelines in multivariate models [16]. In a follow-up report from SEARCH, only 64% reported meeting screening criteria for foot examinations [17]. In addition, those with high HbA_1c_ or hypertension were less likely to receive recommended testing, further propagating risk for adverse outcomes; satisfaction with care was associated with higher likelihood of receipt of recommended HbA_1c_ testing and screening for complications [17] (Table 1).

**Table 1.** Summary of studies examining diabetes mellitus nonadherence to standards of care and increased urgent health-care utilization

Study	Year	Age, y	Key findings	Risk factors for DM nonadherence or urgent health-care utilization	Protective factors for DM adherence and low health-care utilization
Benoit et al [13]	2019	10-64	−Retinopathy screening was poor with 26.3% meeting ADA guidelines	−Decreased eye care referrals	−Eye care education
Bruggeman et al [14]	2021	10-26	−Retinopathy screening occurred on time in only 2/3 of youth with T1D	−Lack of knowledge −Poor geographic access −Transportation difficulties −Depression −Absence of recommendation by health-care provider −Concerns about missed work or school	−Fundal photography in diabetes or primary care clinics with images graded remotely by ophthalmologist −Use of diabetes clinical care coordinators/social workers to address individual barriers
Lake et al [15]	2017	18-39 and 40+	−YA retinal screening behavior influenced by additional cognitive factors compared to older adults	−Stage-of-life constraints −Low perceived personal risk −Unrealistic optimism −Fear of possibility of serious complications −Cost of procedure −Lack of clinician recommendation −Lack of knowledge about screening	−Social influences −Positive reinforcement −Knowledge of connection between DM and eye health
Waitzfelder et al [16]	2011	3-27	−Only 45% of participants aged ≥18 y with T2D reported having their HbA_1c_ levels measured with recommended frequency, compared with 70% of participants aged 12-17 y (*P* = .009)	−Lack of health insurance/gaps in health insurance coverage −Lower family income (annual household income of ≤$25 000)	−Annual family income >$50 000 −Care from pediatric endocrinologists or nurse practitioners/physician assistants
Malik et al [17]	2020	13-21	−64% of youth and YAs with T1D reported meeting screening criteria for foot examinations − 63% reported having urine tested for protein −71% reported having lipid levels checked −81% reported having an eye examination in accordance with ADA recommendations	−Poor clinic attendance	−Greater satisfaction with DM care
Balfe et al [18]	2014	23-30	−Qualitative interviews of young adults with T1D (n = 35) found it difficult to manage DM in workplace due to work-related time pressures and nonroutine nature of work environment	−Nonflexible workplaces	n/a
Ibrahim et al [19]	2021	12-21	−This RCT in youth with T1D showed daily SMS insulin injection reminders for 6 mo led to lower HbA_1c_ 8.8% compared to controls A_1c_ 9.7% (n = 92; 47 controls) (*P* = .03)	n/a	n/a
de Vries et al [20]	2023	≥ 18	−Hospital resource use of Dutch DM patients is high	n/a	n/a
Desai et al [21]	2018	All ages	−Number of DKA-related hospitalizations in United States continues to increase −DKA hospitalizations cost US health-care system $5.1 billion in 2014	−Rate of hospitalizations for DKA higher in men −Rate of increase 2003-2014 of DKA discharges higher in females −DKA discharges in 2014 highest in publicly insured group −Metropolitan areas and southern United States had highest number of DKA discharges	n/a
Wolf et al [22]	2019	18-35	−In a retrospective analysis of 273 YA admitted to an inner-city hospital with DKA or HHS, mean admission A_1c_ was 12.4%. −Main DKA/HHS trigger was medication nonadherence (57.9%) −Only 3.7% used outpatient DM clinics	−Medication nonadherence −Decreased utilization of DM clinics −Lack of health insurance	n/a
Amaize et al [23]	2012	0-29	−Diabetes-related ED visits rates were higher among YAs aged 18-29 y compared with children aged 0-17 y	−Diabetes-related ED visits were higher among patients from lowest-income communities −Public insurance was the most common primary payer in diabetes-related ED visits −Diabetes-related ED visits are higher in micropolitan areas, medium and small metropolitan areas and the Midwest and south United States vs rural and large metropolitan areas and northeast and west United States	
Bronstein et al [24]	2022	19-64	−In this retrospective cohort study of Alabama Medicaid claims data, having a primary care visit in 1 y was significantly associated with reduced likelihood of ambulatory care–sensitive diabetes hospitalization in following year	n/a	−at least one primary care visit in past year
Thind et al [25]	2022	12-29	−This study of ED utilization found that in those ages 18-23 y presence of high-risk conditions (T1D being one of them) was an independent predictor if higher ED utilization	−n/a	−n/a
Yan et al [26]	2018	18-29	−This study looked at ED visits in AYA with T1D and T2D and 30-day outcomes and found 52.5% resulted in hospital admission, 33.8% returned to ED due to hyperglycemia, and 12.5% admitted on subsequent visit	−Most likely precipitants for hyperglycemic ER visits were medication nonadherence, ongoing poor control, infection, and alcohol abuse	−n/a
Feeney et al [27]	2022	≥18	−This study looked at YAs (median age 26 y) admitted to general medicine hospital service with chronic childhood-onset disease, and found T1D was one of the most common underlying chronic diseases in this group (14.6%)	−Hospitalized young adults had low median scores on transition readiness assessment questionnaire and high needs on social determinants of health screening	−n/a

Abbreviations: ADA, American Diabetes Association; AYA, adolescents and young adults; DKA, diabetic ketoacidosis; DM, diabetes mellitus; ED, emergency department; ER, emergency room; HbA_1c_, glycated hemoglobin A_1c_; HHS, hyperglycemic hyperosmolar syndrome; n/a, not available; RCT, randomized controlled trial; SMS, short message service; T1D, type 1 diabetes; T2D, type 2 diabetes; YAs, young adults.

### Between Clinic Care and Coordination

A substantial portion of DM care happens between routine medical visits. Examples of self-care include GM, preventing and treating hypoglycemia, maintaining a healthy diet, engaging in physical activity, scheduling eye and dental care appointments, and inspecting feet regularly for signs of injury or infection. Individuals may need assistance adjusting their insulin doses and reviewing DM sick-day management.

Managing DM self-care in the workplace can be especially difficult for young adults due to time pressure and lack of routine. In one study, young adults shared that their daily variable work schedule made it difficult for them to effectively routinize their DM management practices leading to increased likelihood of skipping, forgetting, or delaying self-management practices [18].

Use of SMS via mobile phones has been studied as an intervention to support young adult patients with DM between clinic visits, but the results are mixed as to whether it improves glycemic control [19].

## Urgent and Emergent Health-Care Utilization

Young adults with DM may have challenges to optimal DM care and regular follow-up, which puts them at risk for acute-care visits to EDs or urgent care facilities, as well as hospitalizations (see Table 1). Hospital costs and resource use for hospitalized patients with DM is high [20]. Hospitalizations for DKA, in particular, seem to be increasing in incidence in the United States and driving up health-care costs, with aggregate national charges for DKA increasing from $2.2 billion to $5.1 billion from 2003 to 2014 (after adjustment for inflation) [21]. Thus, there is clearly a need to investigate ways to reduce hospitalizations in those with DM. In a study of young adults aged 18 to 35 years who were hospitalized for a hyperglycemic emergency, it was found that the main trigger for DKA/hyperglycemic hyperosmolar syndrome (HHS) was medication nonadherence both in T1D and T2D, but more commonly in T1D (61.5% vs 47.9%). Mean HbA_1c_ was high at 12.4%, and only 3.7% attended outpatient DM clinic appointments in the year prior to their admission [22]. This cohort also had a very high rate of ED visits and readmissions in the year following the hospitalization. Thus, high HbA_1c_ and poor clinic attendance are risk factors for hyperglycemic emergencies in the young adult DM population. Visits to the ED were 5 times more likely among young adults ages 18 to 29 years compared to children with DM, and those with uncontrolled DM were 3 times more likely to require hospital admission, most commonly for DKA, when presenting to the ED [23].

Having a primary care visit in the preceding year has been shown to decrease risk of hospitalization in adults with DM [24]. Many young adults may not have a primary care or DM provider during this transitional stage of life, thus putting some young adults at higher risk. T1D has been identified as a health condition associated with high risk of ED utilization in an 18- to 23-year-old cohort of patients in Oregon [25]. A study of young adults ages 18 to 29 years with T1D and T2D found that of those seen in the ED, more than half (52%) resulted in hospital admissions and one-third returned to the ED within 30 days due to hyperglycemia [26]. In addition, 30-day readmission rates are high for young adults with chronic youth-onset diseases such as DM, being 20% in one study of 254 patients, 15% of whom had T1D [27].

## Pregnancy

Pregnancy is another condition that results in health-care utilization among young women with DM. Given the higher risk of adverse pregnancy outcomes compared to women without DM, it is recommended that preconception counseling be incorporated into routine DM care for all women with DM and reproductive potential [28]. However, one study of female adolescents with DM in the SEARCH for Diabetes in Youth study found that only half of them recalled receiving preconception counseling [29]. In a study comparing 12- to 19-year-old adolescents with DM to adolescents without DM, those with DM had an increased risk of adverse pregnancy outcomes as well as higher utilization of outpatient and inpatient care during pregnancy, with payer expenditures increased by 45.3% [30]. A study at a Navajo Area Indian Health service hospital found that while health-care utilization and HbA_1c_ monitoring during pregnancy and immediately post partum was high in women with DM, it was much lower after 8 weeks post partum, with 29% of women having no HbA_1c_ recorded and more than half of the women having no primary care encounter within 2 years post partum [31]. Another study from Ireland found that only 45% of women with T1D and 28% of women with T2D received prepregnancy care, and 22% of all participants were lost to follow-up at 12 months post partum [32]. These studies highlight that while health-care utilization is high during pregnancy for women with DM, this higher level of engagement with health-care teams often does not occur in the critical period prepregnancy, or post partum.

## Mental Health and Diabetes Distress

Mental health comorbidities such as depression, diabetes distress, anxiety, and disordered eating are prevalent in adolescents and young adults with T1D and T2D, and have been shown to clearly affect DM outcomes as well as quality of life [33]. Disordered eating in people with T1D is associated with higher HbA_1c_ and increase in DM complications [34, 35]. Withholding insulin for the purpose of weight loss, “diabulimia,” is an added component that can complicate disordered eating in T1D and contribute to higher HbA_1c_ [36]. People with T2D also have a high prevalence of disordered eating, in particular binge-eating disorder, though association with glycemic control is less clear [37].

Depression and DM distress, which refers to the negative emotional experience from living with the demands of DM, are also both associated with higher HbA_1c_; however, DM distress was found to be a more significant driver for higher HbA_1c_ in adolescents with T1D compared to depressive symptoms [37, 38]. These comorbidities also play a role in health-care utilization and self-management. In one study of patients with T1D, anxiety and depression were associated with lower adherence to self-care [39]. Another study looking at characteristics of young adults with T1D or T2D hospitalized for hyperglycemic emergencies found that 35.5% of these patients had psychiatric comorbidities [22], suggesting that mental health comorbidities are an important risk factor to address when trying to decrease hospitalizations and ED visits in this population.

## Diabetes Technology

Advancements in DM technology in the last few decades have revolutionized the management and monitoring of DM with development of continuous and flash GM, insulin pumps with closed-loop systems, smart insulin pens with Bluetooth connectivity, among others. Technology use has increased from 2010 to 2018, with 7% to 10% more insulin pump use and 5 to 12 times more CGM use in young individuals aged 6 to 26 years [40, 41] However, larger studies such as the T1D Exchange Registry report utilization of DM devices like insulin pumps and CGMs by adolescents and young adults (ages 13-25) is lower than any other age group possibly due to restrictive prescribing patterns by providers and insurance, denying access to patients with high HbA_1c_ [41]. Another possibility is that young adults have difficulty sustaining the use of certain technology such as the hybrid closed loop over time [42]; reasons for discontinuation in one report included financial difficulty obtaining or affording supplies, and sensor issues [43], while another reported “technical issues/problems” such as trouble staying in Auto Mode, perception of “too much work to maintain hybrid closed loop,” and alarm fatigue as common themes shared by hybrid closed loop discontinuers [42].

Telehealth, or remote health-care services delivered through technology, has also been increasingly used since the COVID-19 pandemic in 2020, when it primarily served as a tool for providing medical services while minimizing the risk of virus transmission. Today it continues to be an essential tool that provides medical care for many patients including young adults who have difficulty attending in-person appointments.

Telehealth visits for DM care is feasible because of advances in DM technology with CGMs and insulin pumps that allow individuals to upload their data from home and share it with providers remotely on secure internet-based platforms. Studies on telehealth in young adults with T1D have shown that telehealth increases frequency of follow-up care attendance, reduces physician-related DM distress (eg, “feeling that my doctor doesn't take my concerns seriously enough”) [44], and increases patient care satisfaction [45]. In a randomized controlled trial of young adults with T1D, those with telemedicine visits every 3 months over 1 year had higher attendance of clinic visits compared to controls and compared to the year before the intervention as well as increased adherence to ADA care recommendations [46]. Thus, telemedicine may serve as a valuable tool to keep young adults engaged in their DM care.

## Factors Influencing Health-Care Utilization in Young Adults

### Social Determinants of Health

The Social Determinants of Health (SDOH) encompass the conditions in which people are born, grow, live, work, and age that are influenced by financial stability, power, and resources [47]. These factors have effects at the global, national, and local levels. SDOH include 5 major domains: socioeconomic status, education access and quality, health-care access and quality, neighborhood and environment, and social and community context [47]. While there are data on how SDOH affect health-care utilization in adults with DM [48], there unfortunately is a paucity of data examining young adults (aged 18-25 years) with T1D and T2D. SODH are essential interventional targets to achieve health equity.

### Socioeconomic Status

Socioeconomic status (SES) is a multifactorial concept that includes education, economic, and occupational status. In the United States, 1 in 10 people live in poverty, limiting access to healthy foods, adequate health care, and housing [49]. Having stable employment lowers the risk of living in poverty and increases the likelihood that people can access health care. SES is a strong predictor of T2D onset and progression [48]. Having adequate employment is vital for having adequate financial resources for to pay for health care visits and DM medications and supplies. In the United States, most health insurance is tied to employment. Unfortunately, young adults are more likely to be unemployed or have low-paying jobs compared to older adults, and are the most likely age group to be uninsured [50]. Cost-related nonadherence is high at approximately 20% overall, and close to 30% in uninsured individuals with DM [51]. The high cost of insulin is likely a contributing factor to the rise of DKA observed in young adults with DM over the last decade [51]. Among young adults, 90% endorsed worry about DM costs, and those endorsing concern about DM costs were far less likely to achieve target HbA_1c_ (50% vs 93.6%). There is long-standing evidence that SES is a key determinant of DM outcomes, and the effect of the lack of financial independence and stability on health-care utilization in young adults with DM warrants further study. Food insecurity (FI) can be a metric used to assess SES. The National Health and Nutrition Examination Survey (NHANES) found that 12% of adults with DM were food insecure, and young adults (aged 18-40 years) were more likely to be food insecure than other age groups [52]. For young adults with T1D, the prevalence of FI was 19.5%. Young adults experiencing FI had a 2.3-fold higher odds (95% CI, 1.10-5.09) of high HbA_1c_ and were 2.95 times more likely to have more ED visits compared to young adults with food security [53].

We can extrapolate from adult data the effect of FI on health-care utilization. Adult DM studies demonstrated similar findings of increased health-care utilization, emergency visits, inpatient encounters, avoidable hospitalizations, and significantly higher annual health-care expenditures relative to those who are not food insecure [54] and increased nonadherence to DM medication with an adjusted odds ratio of 23.92 times higher compared to those who were food secure [55].

### Access to Quality Health Care

Having access to adequate health insurance is critical for accessing preventive care and treatment for DM. US Census data from 2022 estimates about 1 in 10 people in the United States do not have health insurance [56]. The landmark implementation of the Affordable Care Act (ACA) expanded access to Medicaid and eliminated preexisting conditions, which resulted in millions of previously uninsured individuals having access to health insurance [57]. The ACA and Medicaid expansion are seen as important tools for increasing access to care and use of preventive services, and affected health-care utilization for young adults with DM. Using a large study cohort of 1371 participants from 2 large national cohorts in the SEARCH and the TODAY study (mean age 25 years [range, 18-36 years]), young adults with T2D were found to have less health-care coverage, and less access to and use of DM care compared to young adults with T1D [58]. Furthermore, less access was associated with higher HbA_1c_. Conversely, state-level Medicaid expansion after the ACA was associated with higher likelihood of health-care coverage and lower HbA_1c_ levels [58]. These are promising data that show access to health-care insurance can lead to better outcomes in young adults with DM; future studies are needed to understand the disparity in health care coverage in youth with T2D.

### Access to Quality Education

People with higher levels of education are more likely to be healthier, better able to navigate the health-care system, and live longer [59]. This is in part due to increased health-care literacy. Patients with DM require self-care skills to manage their disease that are dependent on an individual's ability to collect, comprehend, and follow DM-specific information. In adults aged 20 years and older with T2D, those who have higher health literacy and numeracy (understanding numerical concepts to interpret results from glucometers) had higher medication adherence and lower HbA_1c_ levels [60]. For young adults with DM, their parents have often navigated the health-care system on their child's behalf. During the time of transition the responsibility shifts to the young adult, who now faces the challenges of health-care literacy.

### Neighborhood and Environment

The neighborhoods people live in have a major effect on their health and well-being [61]. Youth living in neighborhoods with high rates of violence and unsafe air or water are at high risk for poor health. Limited access to transportation or living in rural environments with limited health-care access may negatively affect health-care utilization.

Stable housing is another key indicator of economic stability and can influence health-care utilization in people with chronic illness. Housing instability is a spectrum ranging from living in one's car, staying with relatives or friends, suffering evictions or frequent moves, paying more than 50% of income in rent, and living in crowded conditions, to homelessness [62]. Housing instability inherently makes it difficult to prioritize self-care and access health services, which leads to worsening of chronic conditions, exacerbation of complications, and an increase in the use of acute-care services like the ED [63]. The prevalence of housing instability in young adults with DM and whether it affects health-care utilization in the young adult population is unknown. However, what is known is that homelessness is associated with worse DM outcomes in adults [64]. Berkowitz et al [65] examined 1085 adults aged 18 years and older with self-reported DM in the United States and found that 37% self-reported unstable housing. Unstable housing was associated with a greater odds of a DM-related ED visit or hospitalization (adjusted OR 5.17 [95% CI, 2.08-12.87]). Furthermore, they found a generally low use of assistance for social needs, with less than 1% of patients with unstable housing receiving clinic-based assistance. Future studies are needed to understand the prevalence of housing instability and homelessness in young adults with DM and its effect on health-care utilization, and standardizing comprehensive assessment of unmet social needs will enable better service to young adults with DM.

### Social and Community Context

The relationships and interactions with family, friends, and community members can have a considerable effect on a person's health and well-being. Studies of adults with DM have shown that health-care utilization is influenced by sex and racial or ethnic background [66]. Young adults may face challenges related to discrimination based on sex or race, and changes in family structure or social support networks that could limit their ability to access health care.

### Race and Ethnicity

The incidence of both T1D and T2D in youth aged 18 to 25 years is increasing in the United States, particularly among Hispanic and non-Hispanic Black adolescents and young adults [1]. Hispanic adults with DM have higher poverty rates, lower education, and lower physical activity, and are less likely to have insurance coverage with less health-care utilization/expenditures than non-Hispanic adults. This includes fewer outpatient office visits, ED visits, and prescribed medications [67]. To understand if these patterns hold true in young adults with DM, Agarwal et al [4] examined 300 young adults with T1D (aged 18-28 years) across 6 DM centers in the United States and found that non-Hispanic Black and Hispanic young adults had lower SES, higher HbA_1c_ levels, and lower adoption of DM technology compared to non-Hispanic White young adults (*P* < .001). Furthermore, youth with DM in these minority groups had poorer health outcomes compared to non-Hispanic White adults, including high HbA_1c_ levels, increased rates of hospitalization, and increased psychiatric comorbidities and mortality [68]. A large study examining 20 107 children and adolescents with T1D showed that children and young adults of Asian and Pacific Islander individuals had 95% higher odds of in-hospital mortality compared to non-Hispanic White individuals (OR 1.948; 95 CI, 1.015-3.738) [69]. Given these racial and ethnic disparities in health care, novel interventions are necessary to address these gaps in care. Along this vein, Gold et al [70] evaluated the Latino Program (a novel and culturally sensitive shared medical appointment and education program for pediatric Latino patients with T1D) and found it could decrease both ED and hospitalizations in a small study of 57 Latino youth and young adults aged 1 to 20 years with T1D.

### Sex Disparities

Data from the US National Health Interview Survey (2011-2014) examined around 11 500 males and females aged 18 years and older with DM and demonstrated that females reported significantly higher rates of medication nonadherence due to costs and that females were more likely to skip medication, take less medication than prescribed, delay filling prescriptions, and ask doctors to prescribe lower-cost alternative medications [71]. This demonstrates a significant sex-based disparity on medication adherence due to cost. Similar sex differences were seen in Japan, with adult men having higher adherence to T2D medications than women [72]. The T1D Exchange also identified an increased rate of DKA (5% vs 3%; *P* < .001) in women compared to men older than 18 years with T1D [73]. Furthermore, there are combined racial and ethnic disparities in DM care in women. Marshall et al [74] examined 2016 data from Kaiser Permanente and looked at women aged 18 to 44 years with T1D or T2D (n = 9923); 83% had T2D. One-third of women with T2D had nonadherence to DM medications. Black and Hispanic women with T2D and T1D were more likely to have an HbA_1c_ greater than 9%.

### Household Structure

Major life events such as parental divorce or moving to a new home may act as stressors that could negatively affect young adults, and the effect could be amplified for young adults with DM. A small study evaluated how major life events (such as hospitalization of a family member, serious illness/injury in a family member, or death of a family member) affected DM management in youth with T1D. Youth with T1D experienced on average 2.5 ± 2.7 events/year. Teens experiencing more than 4 events had significantly poorer adherence, lower self-efficacy for managing DM, and poorer quality of life compared to teens with fewer events [2]. Studies are needed in young adults with T1D and T2D, particularly given their high likelihood of changes in household structure.

## Conclusion

**Figure 2.**
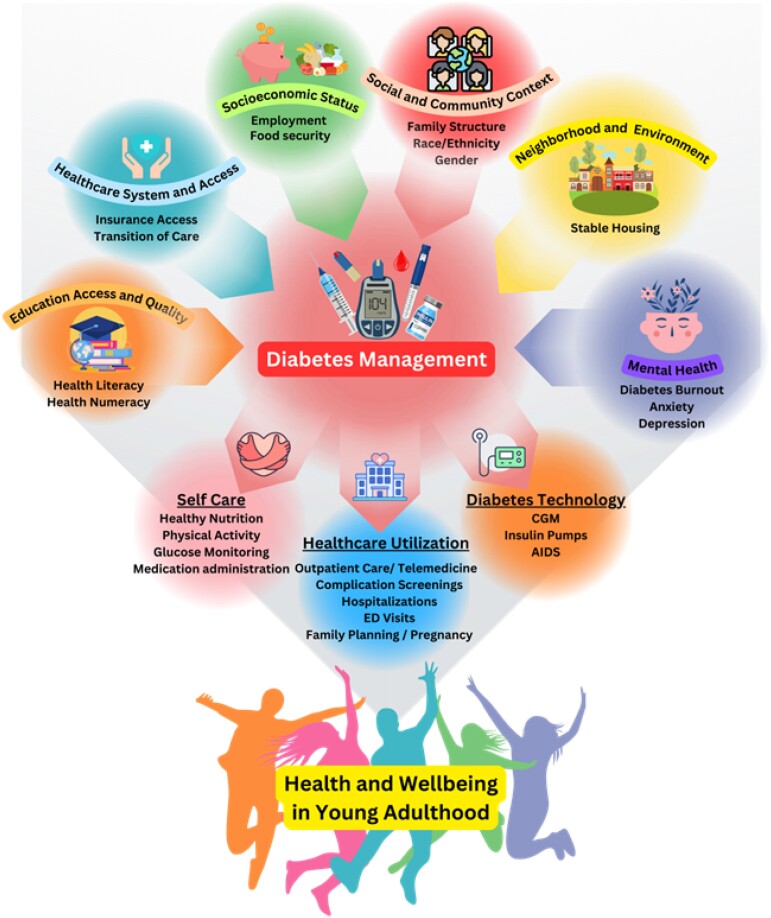
Factors influencing diabetes management and health outcomes in young adults: Young adults with diabetes mellitus (DM) face several internal and external factors that influence their health management, including education, health-care systems and access, socioeconomic status, social and community contexts, the neighborhood and environment, as well as mental health. These factors influence a young adult's ability for DM self-care, ability to access health care, and use of DM technologies. AIDs, automated insulin delivery systems; CGM, continuous glucose monitoring; ED, emergency department.

Young adulthood is a high-risk period for individuals with DM as they experience increasing independence and life transitions that can make it difficult to engage in DM care. Compared to older adults, young adults are more likely to have an HbA_1c_ greater than 9% and less likely to have the recommended HbA_1c_ target of less than 7% [3]. In addition, they have decreased adherence to standards of care in DM, and high utilization of ED visits and hospitalizations for DKA. Contributing factors may include poor transition from pediatric to adult care leading to gaps in care and treatment, lack of financial stability contributing to cost-related nonadherence, increased prevalence of mental health comorbidities, and decreased attendance of primary care and DM clinic visits [22]. Research focusing on improving transitional care, DM technology, and the effect of social determinants of health such as SES, education access and quality, health-care access and quality, neighborhood and environment, and social and community context (Fig. 2) on young adults with DM are critically needed so that targeted interventions can be developed to reduce acute-care utilization and improve health-care outcomes.

## Data Availability

Data sharing is not applicable to this article as no data sets were generated or analyzed during the current study.

## Abbreviations

ACA: Affordable Care Act
ADA: American Diabetes Association
CGM: continuous glucose monitor
DKA: diabetic ketoacidosis
DM: diabetes mellitus
ED: emergency department
FI: food insecurity
HbA_1c_: glycated hemoglobin A_1c_
HHS: hyperglycemic hyperosmolar syndrome
ISPAD: International Society for Pediatric and Adolescent Diabetes
LEAP: Helmsley T1D Transition Let's Empower and Prepare
SDOH: Social Determinants of Health
SES: socioeconomic status
SMS: short message service
T1D: type 1 diabetes
T2D: type 2 diabetes
